# Re-evaluation of mouse models of endometriosis for pathological and immunological research

**DOI:** 10.3389/fimmu.2022.986202

**Published:** 2022-11-18

**Authors:** Ying He, Bo Liang, Sze Wan Hung, Ruizhe Zhang, Hui Xu, Jacqueline Pui Wah Chung, Chi Chiu Wang

**Affiliations:** ^1^ Department of Obstetrics and Gynaecology, The Chinese University of Hong Kong, Hong Kong, Hong Kong SAR, China; ^2^ Department of Gynecological Oncology, Cancer Hospital and Shenzhen Hospital, Chinese Academy of Medical Sciences and Peking Union Medical College, Shenzhen, China; ^3^ Li Ka Shing Institute of Health Sciences, The Chinese University of Hong Kong, Hong Kong, Hong Kong SAR, China; ^4^ School of Biomedical Sciences, The Chinese University of Hong Kong, Hong Kong, Hong Kong SAR, China; ^5^ Chinese University of Hong Kong, Sichuan University Joint Laboratory in Reproductive Medicine, The Chinese University of Hong Kong, Hong Kong, Hong Kong SAR, China

**Keywords:** endometriosis, animal models, pathology, immunology, surgical transplantation

## Abstract

Endometriosis is an estrogen-dependent gynecological disease with chronic pelvic inflammation. In order to study the pathophysiology of endometriosis and examine the therapeutic effects of new pharmaceuticals for endometriosis treatment, different animal models had been developed in the last two decades, especially mouse models. However, no study evaluated the effects of various modeling approaches on pathology and immunology in endometriosis. This study aimed to compare endometriotic lesion development and immune profiles under different methods of establishing endometriosis models in mice, including estrus synchronization (ovariectomy with estrogen supplement versus male urine-soaked transfer bedding), endometrium preparations (whole uterus including endometrium and myometrium fragments versus solely endometrium fragments), and surgical transplantation (subcutaneous transplantation versus intraperitoneal injection). Our results showed that lesion growth under estrus synchronization by ovariectomy with estrogen supplement had a higher success rate and more proliferative endometrium, apart from higher body weight gain. Immune responses in peripheral blood were similar in the whole uterus and solely endometrium fragments and in intraperitoneal injection and subcutaneous transplantation, but a more innate immune response in the peritoneal microenvironment was found in solely endometrium fragments and intraperitoneal injection than counterparts. In conclusion, different endometriosis modeling methods result in different pathological and immunological features. Ovariectomy with estrogen supplement, solely endometrium fragments, and intraperitoneal injection are more suitable for both pathological and immunological studies of endometriosis in mice, which are important for mechanistic studies and immunotherapy development.

## Introduction

Endometriosis is a common but complex benign gynecological disorder characterized by the presence of endometrial glands and stroma outside the uterine cavity. It is one of the main causes of pelvic pain and infertility in reproductive women. There is no cure treatment or targeted therapy available so far. Commonly used hormonal therapies can only relieve the pain and ease the symptoms, while conservative surgery cannot thoroughly remove the endometriotic deposits. In addition to the unpleasant side effects, the recurrence rate of the treatment is high (1). Non-steroidal anti-inflammatory drugs may cause gastric irritation and peptic ulcers when frequently used. Long-term use may further result in kidney damage in form of renal papillary necrosis and even organ failure. Side effects followed by combined oral contraceptive pills include heavy uterine bleeding, peripheral edema, decreased libido, nausea, weight gain, paraesthesia, breast tenderness, insomnia, and headache. Progestins induced breakthrough bleeding, weight gain, edema, acne, breast tenderness, headaches, bone loss, mood changes, and muscle cramps.

Animal models allow us not only to study the fundamental mechanisms of endometriosis but also to test new pharmaceuticals for potential treatment. Endometriosis develops spontaneously only in species that have menstruation; it occurs in humans and non-human primates (2). However, the cost and ethical concerns limit the use of non-human primates on a large scale, so rodents (including mice, rats, and hamsters) and non-rodents (such as rabbits) have been used to establish endometriosis models; among those, mice and rats have been widely used in recent years (3). Endometriosis mouse models can be induced by transplanting endometrial tissue either subcutaneously (S) (4) or intraperitoneally (IP) (5). The intraperitoneal mouse model resembles the human endometriosis microenvironment, and it is widely used in immunological studies, but we could not monitor lesion growth and development in that model non-invasively. The subcutaneous mouse model is convenient for monitoring lesion development on the abdominal surface, and it is often used in pharmaceutical research, but whether it resembles the immune response of the abdominal cavity has not been studied. For the syngeneic mouse endometriosis model, the used implanted tissues included either whole uterus tissue (W) (6) or isolated endometrium fragments or cells (Em) (7). Whether the content and quantity of the implanted endometrium influence the degree of the immune response has also rarely been studied. Different estrus cycle stages may result in different outcomes in endometriosis growth and development as well (8). Estrus synchronization can be achieved by ovariectomy (OVX) followed by estrogen supplement (E2) or male urine-soaked transfer bedding (TB), and whether these methods have different impacts on endometriosis development was not fully understood before.

Endometriosis is characterized by recurrent ectopic endometrium proliferation and chronic pelvic inflammation (9). However, there is still no study that evaluated the effects of different modeling approaches on the pathology and immunology in endometriosis. There is no systematic evaluation of different estrus synchronization, endometrium preparations, and surgical transplantation. Whether different approaches may have different outcomes in the endometriosis model are still unknown. Therefore, we compared different estrous cycle synchronization methods. First, we observed the endometriotic lesions morphologically through hematoxylin and eosin (H&E) staining; then, we detected apoptosis and proliferative activity by immunohistochemical staining. However, it was hypothesized that women with endometriosis have defective immune responses; therefore, their immune systems are not able to recognize and carry out proper defense against the endometrial deposits in the ectopic site (9). This promotes the spread of endometrial cells and favors angiogenesis and tissue adhesion, as well as induces inflammation (10). Neutrophils and macrophages do participate in the initiation of endometriosis and elevate pro-inflammatory cytokines to recruit other immune cells like myeloid-derived suppressor cells (MDSCs) to escape immunosurveillance of natural killer (NK) and dendritic cells. Other studies also showed a decrease in the cytotoxicity of NK cells and an enhancement in the activation of peritoneal macrophages in endometriosis (11) (12). Recently, immunotherapy has become a new trend in the treatment of endometriosis (9). Immunosuppressive cells, such as neutrophils N2, mast cells, macrophage M2, induced dendritic cells (iDC), Regulatory T cells (Tregs), Th17, regulatory B cells (Breg), MDSCs, and immunosuppressive cytokines, including IL-4, IL-10, IL-13, TGFα, and growth factors, form an immunosuppressive network and thereby play a critical role in peritoneal implantation, angiogenesis, and local proliferation of endometrial cells. Immunotherapy targets on key functional immune cells, cytokines, and molecular signaling pathways provide potential strategies for treatment of endometriosis.

This is a comparative study that aimed to evaluate the effects of different modeling approaches on pathology and immunology in endometriosis. Here, we also focused on the impact of synchronization on the development of the endometriotic lesions and transplantation tissues and locations on immune response in the endometriosis models. The results of the study identified optimal modeling for mechanistic studies and immunotherapy development for endometriosis research.

## Materials and methods

### Study designs

**Figure 1:**
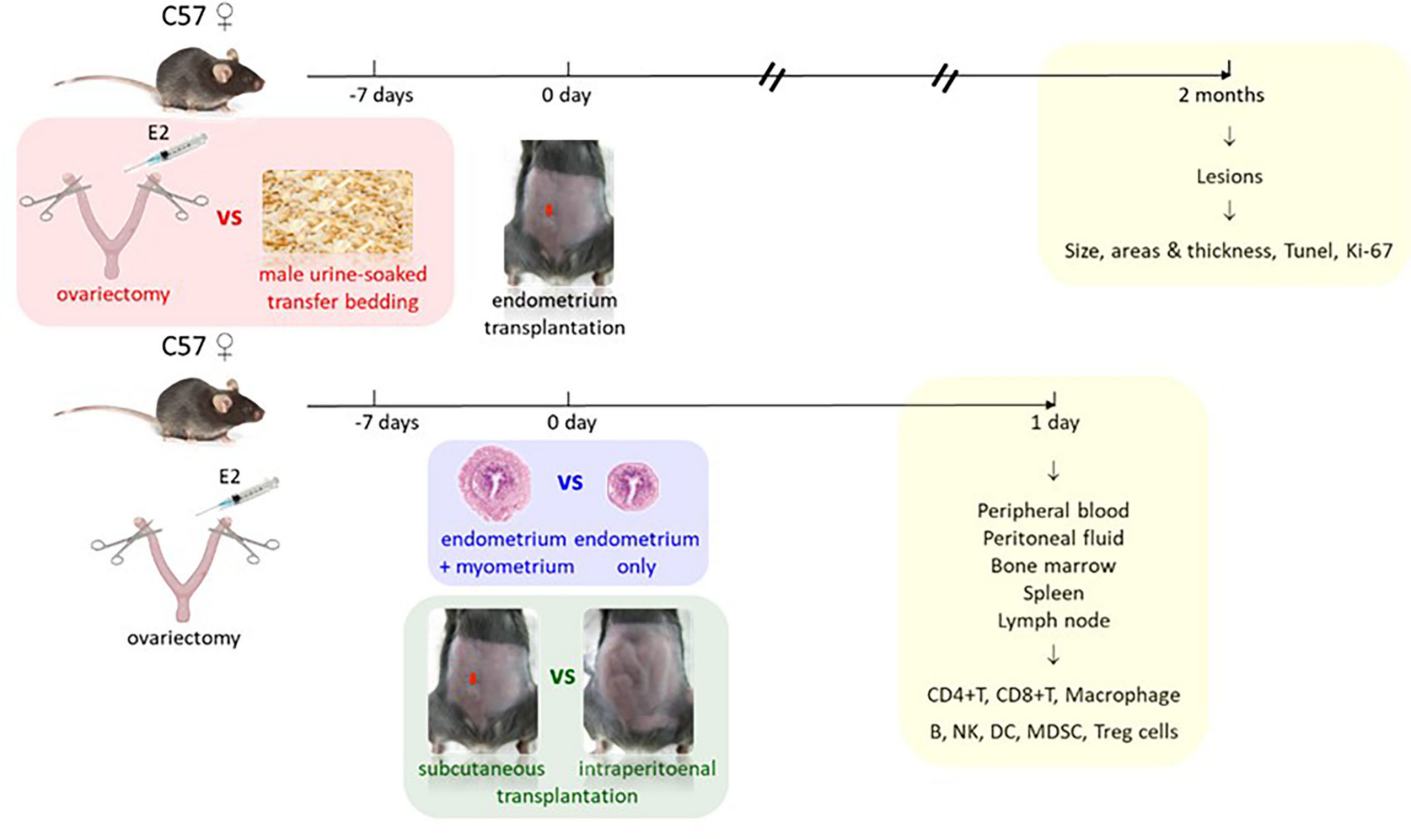
Study design. Comparison 1 (red): estrus synchronization by ovariectomy with estrogen supplement (E2) versus male urine-soaked transfer bedding with endpoint 2 months after transplantation for pathological studies (yellow). Comparison 2 (blue): endometrium preparations by whole uterus including endometrium and myometrium versus solely endometrium. Comparison 3 (green): surgical transplantation by subcutaneous (red arrow) versus intraperitoneal transplantation with endpoint 1 day after transplantation for immunological studies (yellow).

Endometriosis growth and development and its immune responses under different methods were evaluated by comparing different endometriosis models in mice, including estrus synchronization, i.e., OVX with E2 versus TB; endometrium preparations, i.e., whole uterus including endometrium and myometrium fragments (W) versus solely endometrium fragments (Em); and surgical transplantation, i.e., subcutaneous transplantation (S) versus intraperitoneal injection (IP), for pathological and immunological studies (**Figure 1**). For pathological studies, the endometriotic lesions needed time to develop, the size of the endometriotic implants in mice reached the maximum in the fourth week, and the implants continued to grow in the eighth week (13), so the animals were sacrificed later 2 months after transplantation. For immunological studies, white blood cells were recruited to the peritoneal cavity during the early initiation of endometriosis in mice, they increased transiently in 24 and 48 h, and by 72 h, the cells decreased (14). Based on this study, we decided to study the early immune profiles for 24 h, so the animals were sacrificed earlier at 1 day after the transplantation.

### Animals

C57BL/6 female mice aged 6–8 weeks were provided by the Laboratory Animal Service Center of the Chinese University of Hong Kong and maintained in a pathogen-free animal house in Prince of Wales Hospital. The mice were kept in 12 to 12 h of regulated light–dark cycle and ambient temperature at 21°C–22°C with humidity of 60%. All the mice had free access to chow and tap water and were acclimated at least for a week before experiments. All animal work was approved by the Animal Experimentation Ethics Committee of the Chinese University of Hong Kong and performed under ethics 18-222-MIS in accordance with international standards and guidelines. Five to eight mice were included in each group and experiment for comparisons.

### Ovariectomy with estrogen supplement versus male urine-soaked transfer bedding

Estrus cycle synchronization was applied in two different ways at random: OVX+E2 (15) and TB (16). We used cytological evaluation of vaginal smears of the female mice to monitor the estrous cycle stage (17). The vaginal opening appearance of mice in the proestrus phase was characterized by swollen, moist, and pinkish tissue. It was usually wrinkled or striped along the dorsal and ventral margins. When mice entered the estrus phase, the vaginal opening became less pink, moist, and swollen. The vaginal opening in the metestrus phase was constricted, and white cellular debris was sometimes visible. During the diestrus phase, the vaginal opening was small and closed, and there was no tissue swelling. For the OVX+E2 group, mice were first subjected to OVX (a week before transplantation) and intramuscularly (i.m.) injected with 100 μg/kg of estradiol-17β (E2) (Sigma, St. Louis, MO, USA; E2758) dissolved in oil at the time of OVX and also at the fifth day after OVX (18, 19). In the TB group, mice were transferred to urine-soaked male bedding once every 5 days and at least one cycle before transplantation. At day 0 of transplantation, donor mice were sacrificed to remove the uterus, and the removed uterus was kept in ice-cold phosphate-buffered saline (PBS), diluted from 10× PBS (Invitrogen, Carlsbad, CA, USA; AM9625). Fat tissues were then removed. The uterine horns were opened longitudinally with a small scissor and divided into equal-sized implants with a 2-mm sterile biopsy punch in the petri dish. The endometrial fragments were transplanted subcutaneously by inserting lesions surgically into subcutaneous pockets created by abdominal wall incisions along the belly. The abdominal incision was closed with a 5-0 surgical silk suture (Johnson & Johnson, New Brunswick, NJ, USA; W500H). Lesion sizes were measured by caliper on the abdominal surface every 3 days. After the transplantation, the body weight in each mouse of pathological study was measured every 6 days. The mice were sacrificed at 2 months after the transplantation. Endometriotic lesions were dissected out and washed in sterile PBS; then, all the lesions were fixed with 4% formalin (Sigma), dehydrated through graded ethanol, cleared in xylene, and embedded in paraffin wax for histological analysis.

### Subcutaneous transplantation versus intraperitoneal injection, and whole uterus including endometrium and myometrium fragments versus solely endometrium fragments

We also compared the endometriosis transplantation models by IP with those by S and monitored immune cell changes in early endometriosis development. Based on the success rate of estrus synchronization from the pathological studies, OVX supplemented with E2 methods was employed in order to eliminate individual variation and ensure stable E2 levels in the animals for the immunological studies. Both donor and recipient mice were subjected to OVX and i.m. injected with E2 as described above. One week after OVX, the uterine horns from the donor mice were removed into a sterile PBS medium. For the W group, the whole uterus was used. For the Em group, the serosa and myometrium were peeled off under a microscope. Then, the tissue fragments were obtained with a 2-mm biopsy punch; 35 mg Em or W was suspended in 0.3 ml of sterile PBS in a sterilized injection syringe and then either injected into the peritoneal cavity of recipient mice with an 18-G needle (20) as the IP group or surgically transplanted in a subcutaneous pocket as the S group. For the control group (CIP and CS), they underwent OVX+E2 and sham surgeries, and 0.3 ml of sterile PBS was used. All the mice in the immunological study were sacrificed 1 day after implantation. The peritoneal cavity, bone marrow, blood, spleen, and lymph node were collected for flow cytometry analysis.

### Hematoxylin and eosin staining

After embedding, 4-μm serial paraffin sections of the lesions were prepared on coated slides. H&E staining was carried out in the middle section of the lesion. Staining procedures were the same as described before (21). After H&E staining, we measured different parameters, including lesion area and thickness in each section. The lesion area was calculated by ImageJ. The average endometrium thickness was calculated by the formula as endometrium thickness index: ETI = 2A/(P1 + P2), where A is π, and P1 and P2 are the area of the endometrium and uterus cavity, respectively (22).

### Immunohistochemistry staining and TUNEL assay

The proliferation of the lesions was assessed immunohistochemically using Ki-67, a monoclonal antibody against a nuclear antigen present only in proliferating cells (23). Tissue sections were deparaffinized using xylene and then rehydrated in serial concentrations of ethanol and tap water. Antigen retrieval in sodium citrate buffer was induced by heating the sections on the slides with buffer in a microwave for 20 min. Hydrogen peroxide solution (30% H_2_O_2_ in 100% methanol) was used to inactivate endogenous peroxidase. Slides were incubated with either primary antibody (rabbit anti-mouse Ki-67 antibody, D3B5, Cell Signaling Technology, Danvers, MA, USA) or PBS as a negative control at 4°C overnight and then washed. The secondary antibody (donkey anti-rabbit IgG-HRP, sc2313, Santa Cruz, Dallas, TX, USA) was applied, and the slides were incubated for 1 h at room temperature. Color was developed using a DAB kit (k3468, Dako, Glostrup, Denmark). The sections were counterstained slightly with hematoxylin and dehydrated in a serial concentration of ethanol, cleared in xylene, and mounted with coverslips before microscopic examination and imaging.

*In situ* end-labeling of fragmented DNA (TUNEL) assay was performed to identify cells involved in the apoptotic fragment of DNA (24). Apoptotic cells in lesions were examined using a TUNEL kit (ApopTAG^®^, S7100, Millipore, Billerica, MA, USA) according to the manufacturer’s instructions. The numbers of Ki-67- and TUNEL-positive cells in the epithelium and stroma were quantified separately. The numbers of labeled cells per ×400 microscopic fields were counted manually by two trained observers who were blinded to the experiments and grouping. For each section, at least five high-power microscopic fields in each sample were selected randomly for analysis as previously described (25).

### Flow cytometry

Recipient mice were sacrificed by cervical dislocation at 24 h after transplantation of endometrial fragments for sample collection. Cells from the peritoneal cavity, bone marrow, blood, spleen, and lymph node were included and collected. Peritoneal cells were collected by injecting ice-cold PBS [with 5% fetal bovine serum (FBS)] into the peritoneal cavity (26). Whole blood samples were collected *via* cardiac puncture (27). Muscles around the legs were removed, and femoral bones were cut at the knee joint. Bone marrow cells were expelled from the end of the bone by flushing with ice-cold PBS using a 1-ml syringe with a 25-G needle (28). A single cell suspension was prepared by first cutting the spleens and lymph node into several pieces and then homogenizing the pieces using the top of a syringe as a plunger and then rinsing through the cell strainer (29). The collected cells from different samples were centrifuged for 10 min at 4°C, followed by red blood cell lysis (RBC lysis buffer; BioLegend, San Diego, CA, USA; 420301). Cell pellet was resuspended in 5% FBS ((Life Technologies, Carlsbad, CA, USA; 10270106) in PBS buffer for flow cytometry analysis.

**Figure 2:**
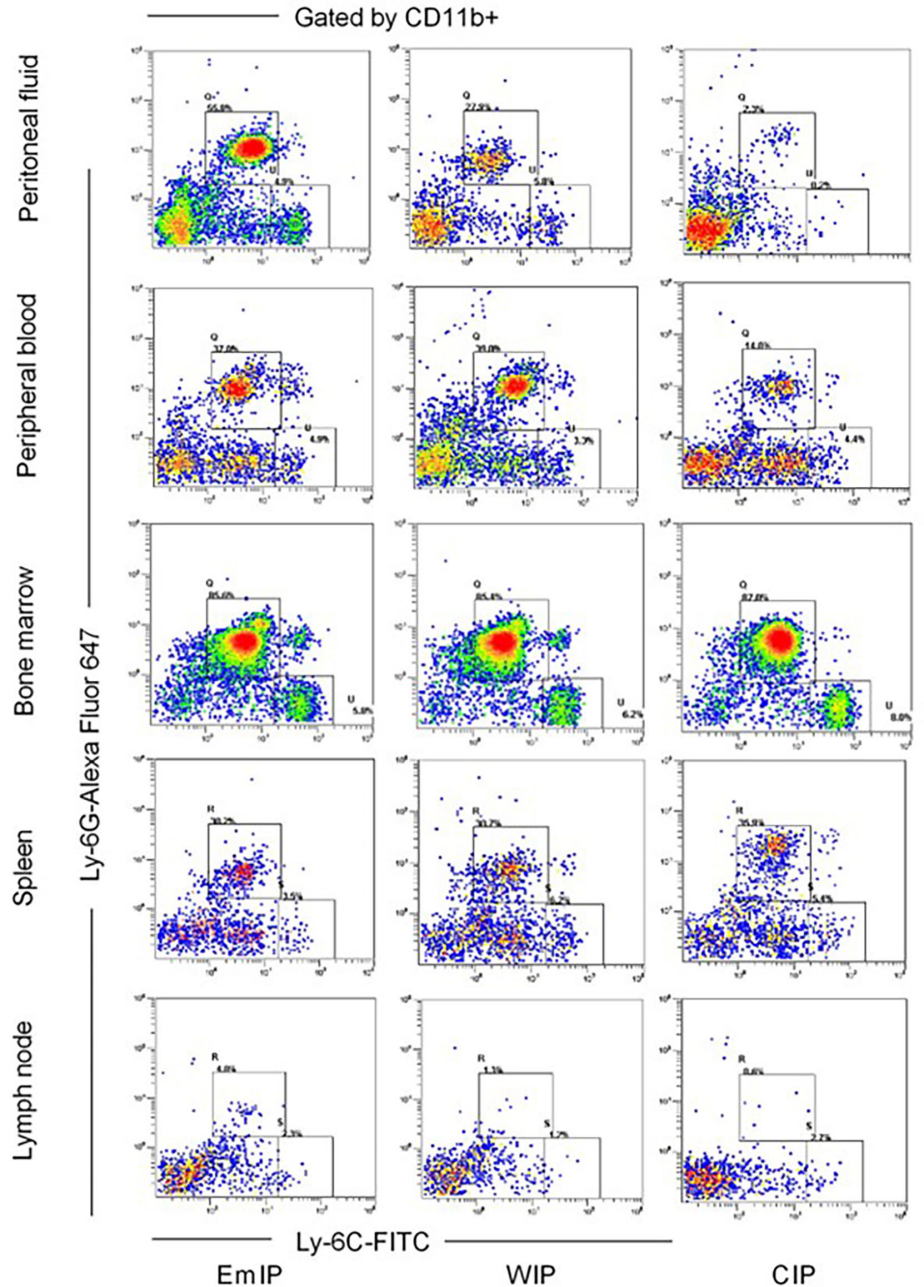
Gating strategy for identification of mouse MDSC subsets in peritoneal fluid, peripheral blood, bone marrow, spleen, and lymph node of endometriosis mice from OVX+E2 model. After exclusion of doublets (not shown), live CD11b+ cells were gated, and the proportion of Ly6G and Ly6C cells was evaluated. G-MDSC (CD11b+ Ly6G+ Ly6Clo) and M-MDSC (CD11b+ Ly6G-Ly6Chi). EmIP, endometrium fragments and intraperitoneal; WIP, whole uterus tissue and intraperitoneal; CIP, sham control and intraperitoneal; G-MDSC, granulocytic myeloid-derived suppressor cells; M-MDSC, monocytic myeloid-derived suppressor cells.

Immune cells in different samples were counted by flow cytometry analysis using FC 500 cytometer (Beckman Coulter, Brea, CA, USA). A total of 1 × 10^6^ cells were incubated with primary antibodies, including CD3-FITC (BD Biosciences, San Jose, CA, USA; 553062) for CD3+ T cells, CD4-PE-cy5 (BD, 553050) for CD4+ T cells, CD8-PE (BioLegend, 100708) for CD8+ T cells, CD11b-PE/Cy7 (BioLegend, 101216), F4/80-PerCP/Cy5.5 (BioLegend, 123128) for macrophages, CD19-FITC (BioLegend, 115506) for CD19+B cells, CD49b-FITC (BD, 553857) for NK cells, CD11b-Alexa Fluor 647 (BioLegend, 557686), CD11c-PE/Cy7 (BioLegend, 117318) for dendritic cells, CD11b-PE/Cy7 and Ly-6G-Alexa Fluor 647 (BioLegend, 127610), and Ly-6C-FITC (BioLegend, 128006) for MDSCs for 30 min at 4°C. Intracellular staining for Treg cells was carried out by True-Nuclear™ Transcription Factor Buffer Set (BioLegend, 424401) with an antibody against FOXP3-PE (BioLegend, 126404). Then, the stained cells were washed and resuspended in 1 ml of staining buffer for further analysis. Data obtained were analyzed by CXP Analysis software. The gating strategy for the identification of mouse MDSC subsets is shown in **Figure 2**.

### Statistical analysis

Quantitative data were expressed as mean ± SEM. Statistical analysis was carried out by using the software SPSS 21. Independent t-test or analysis of variance (ANOVA) was used to determine the statistical changes, followed by post-hoc comparisons of individual groups using the Bonferroni correction, when appropriate. Differences were considered significant at a level of p < 0.05.

## Results

### Lesion growth and development

**Figure 3:**
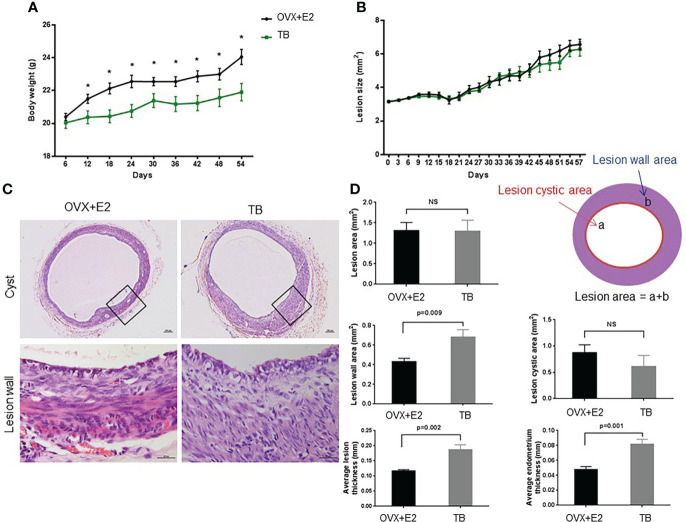
Dynamic changes of **(A)** body weight and **(B)** lesion size after inducing endometriosis. OVX+E2, ovariectomy with estrogen supplement group; TB, male urine-soaked transfer bedding group. **(C)** Representative hematoxylin and eosin-stained images of endometriotic lesions from mouse model of endometriosis at 2 months post-induction. Cyst scale bar, 100 μm. Lesion wall scale bar, 25μm. **(D)** Endometriotic lesions growth parameters post-induction. Data are shown as mean ± SEM, n = 8, for each group. * p < 0.05 by independent t-test or ANOVA. NS, no significance.

**Figure 4:**
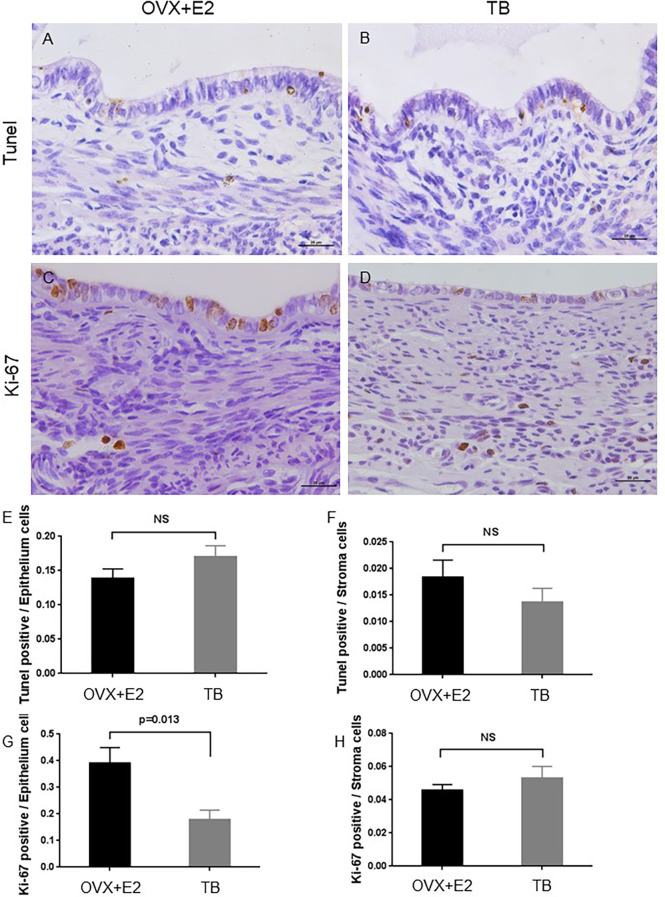
Representative images of TUNEL assay and Ki-67-stained sections of endometriotic lesions from mouse model of endometriosis at 2 months post-induction. OVX+E2, ovariectomy with estrogen supplement group; TB, male urine-soaked transfer bedding group. **(A)** TUNEL assay of endometriotic lesion from OVX+E2 group. **(B)** TUNEL assay of endometriotic lesion from TB group. **(C)** Ki-67 staining of endometriotic lesion from OVX+E2 group. **(D)** Ki-67 staining of endometriotic lesion from TB group. Scale bar, 25μm. **(E)** TUNEL-positive cells per epithelium cells. **(F)** TUNEL-positive cells per stroma cells. **(G)** Ki-67-positive cells per epithelium cells. **(H)** Ki-67-positive cells per stroma cells. Data are shown as mean ± SEM of the ratios, n = 8, for each group. Statistics by independent t-test. NS, no significance.

From day 12 after transplantation (day 0), body weight was significantly increased in the OVX+E2 group when compared to the TB group (**Figure 3A**). Dynamic changes of lesion size are shown in (**Figure 3B**). Representative H&E images of endometriotic lesions from mouse model of endometriosis at 2 months post-induction are shown in **Figure 3C**. The transplantation success rate (number of lesions grew into endometriotic lesions/number of lesions transferred) of the OVX+E2 group (73.9%) was higher than that of the TB group (54.2%). The lesions grew steadily for 2 months, and they eventually developed into fluid-filled, round structures composed of endometrium and myometrium tissues. No significant differences were found in lesion size and lesion cystic area between the two groups (OVX+E2 group *vs.* TB group) (**Figure 3D**). However, lesion wall area (0.419 ± 0.032 *vs.* 0.682 ± 0.076 mm^2^), average lesion thickness (0.117 ± 0.005 *vs.* 0.187 ± 0.017 mm), and average endometrium thickness (0.048 ± 0.004 *vs.* 0.082 ± 0.007 mm) were significantly smaller in the OVX+E2 group than in the TB group (**Figure 3D**). However, the OVX group had significantly higher Ki-67-positive cell counts in epithelium cells than the TB group (**Figure 4**), suggesting a higher successful rate of endometriosis modeling and proliferative activity in the epithelium cells of the OVX+E2 group.

### Immunological changes

**Figure 5:**
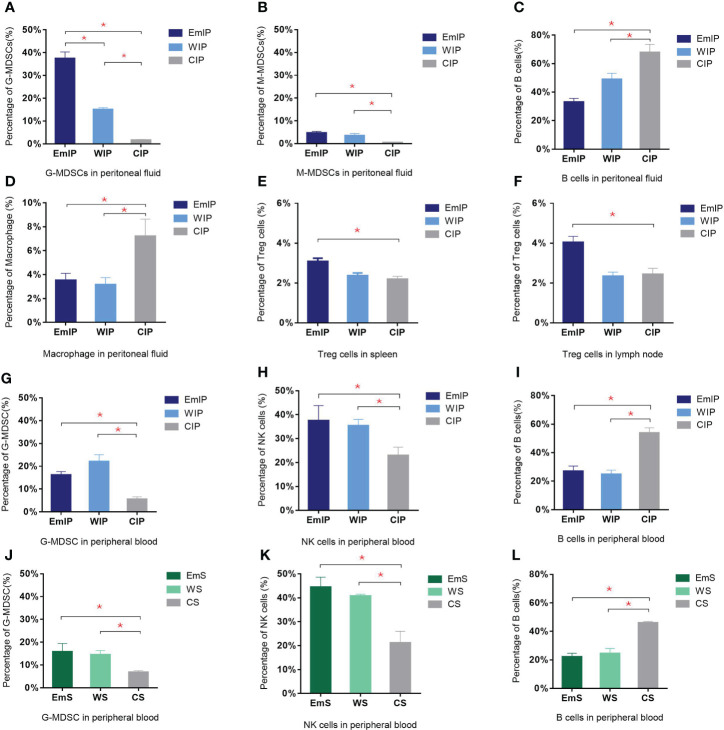
Changes in immune cells in peritoneal fluid, peripheral blood, spleen, and lymph node of endometriosis mice. There were significant increases in both granulocytic and monocytic myeloid-derived suppressor cells (G-MDSC and M-MDSC) but a decrease in B cells and macrophages in intraperitoneal injection group in peritoneal fluid **(A–D)**. Increase in G-MDSC and NK cells and decrease in B cells were found in both intraperitoneal injection **(G–I)** and subcutaneous transplantation **(J–L)** groups in peripheral blood. Significant increase in Treg cells in endometrium intraperitoneal injection group in spleen **(E)** and lymph node **(F)**. IP, intraperitoneal injection; S, subcutaneous transplantation; EmIP, endometrium intraperitoneal injection. Data are shown as mean ± SEM, n = 5, for each group. * p < 0.05 by ANOVA. EmIP, endometrium fragments and intraperitoneal; WIP, whole uterus tissue and intraperitoneal; CIP, sham control and intraperitoneal; EmS, endometrium fragments and subcutaneous; WS, whole uterus tissue and subcutaneous; CS, sham control and subcutaneous; G-MDSC, granulocytic myeloid-derived suppressor cells; M-MDSC, monocytic myeloid-derived suppressor cells.

In peritoneal fluid, B cells were significantly decreased in the EmIP and WIP groups when compared to the control (EmIP, 0.330% ± 0.026%; WIP, 0.490% ± 0.042%; CIP, 0.679% ± 0.057%) (**Figure 5C**). G-MDSC and M-MDSC were significantly increased when compared to the control (G-MDSC: EmIP, 0.374% ± 0.029%; WIP, 0.151% ± 0.008%; CIP, 0.016% ± 0.003%; M-MDSC: EmIP, 0.047% ± 0.007%; WIP, 0.035% ± 0.009%; CIP, 0.005% ± 0.001%; **Figures 5A, B**). In contrast, macrophage was slightly decreased in the EmIP and WIP groups when compared to the control (EmIP, 0.035% ± 0.060%; WIP, 0.032% ± 0.006%; CIP, 0.072% ± 0.014%; **Figure 5D**). The EmS and WS groups had no significant changes in immune cells in peritoneal fluid when compared to the control. In peritoneal fluid, G-MDSC and M-MDSC in the IP group were significantly higher than in the S group (G-MDSC: EmIP, 0.374% ± 0.029%; WIP, 0.151% ± 0.008%; CIP, 0.016% ± 0.003%; M-MDSC: EmIP, 0.047% ± 0.007%; WIP, 0.035% ± 0.009%; CIP, 0.005% ± 0.001%; G-MDSC: EmS, 0.057% ± 0.016%; WS, 0.043% ± 0.009%; CS, 0.045% ± 0.007%; M-MDSC: EmS, 0.021% ± 0.004%; WS, 0.019% ± 0.003%; CS, 0.018% ± 0.002%).

In peripheral blood, B cells were significantly decreased in the EmIP and WIP groups when compared to the control (EmIP, 0.270% ± 0.036%; WIP, 0.249% ± 0.029%; CIP, 0.538% ± 0.036%; **Figure 5I**). G-MDSC significantly increased (EmIP, 0.162% ± 0.015%; WIP, 0.221% ± 0.030%; CIP, 0.056% ± 0.010%; **Figure 5G**). In contrast, NK cells were significantly increased in the EmIP and WIP groups when compared to the control (EmIP, 0.375% ± 0.063%; WIP, 0.354% ± 0.026%; CIP, 0.230% ± 0.035%; **Figure 5H**). B cells were also significantly decreased in the EmS and WS groups in peripheral blood when compared to the control (EmS, 0.221% ± 0.025%; WS, 0.245% ± 0.035%; CS, 0.460% ± 0.010%; **Figure 5L**). G-MDSC was significantly increased (EmS, 0.159% ± 0.036%; WS, 0.145% ± 0.018%; CS, 0.068% ± 0.007%; **Figure 5J**). In contrast, NK cells were significantly increased in the EmS and WS groups when compared to the control (EmS, 0.445% ± 0.042%; WS, 0.407% ± 0.008%; CS, 0.212% ± 0.048%; **Figure 5K**). G-MDSC and M-MDSC have no significant difference between the IP and S groups in peripheral blood.

In the bone marrow, there were no significant changes in different immune cells in the EmIP or WIP versus CIP group and the EmS or WS versus CS group. In the spleen and lymph node, Treg cells were significantly increased in the EmIP group when compared to the WIP and CIP groups (spleen: EmIP, 0.031% ± 0.002%; WIP, 0.024% ± 0.001%; CIP, 0.022% ± 0.001%; lymph node: EmIP, 0.040% ± 0.003%; WIP, 0.023% ± 0.002%; CIP, 0.024% ± 0.003%; **Figures 5E, F**). The EmS and WS groups had no significant immune cell changes in peritoneal fluid, bone marrow, spleen, and lymph nodes. Summary of experiments and results are shown in **Table 1**.

**Table 1:** Summary of experiments and results.

Study	Estrus	Engraft Tissue	Transplantation	Duration	Outcome measures and results				
	synchronization								
	** **	** **	** **	** **	Leison size	Leison area	Thickness	Endometrium apoptosis by TUNEL	Endometrium proliferation by Ki67
Pathology	ovariectomy with estrogen supplement;	endometrium	subcutaneous	2 months	NS	wall ↓	lesion & endometrium ↓	epithelium & stroma NS	epithelium ↑, stroma NS
	male urine-soaked transfer bedding				–	–	–	–	–
Immunology					Bone marrow	Spleen	Lymph nodes	Peritoneal blood	Peritoneal fluid
	ovariectomy with estrogen supplement	endometrium	subcutaneous	1 day	NS	NS	NS	B cells ↓, G-MDSC & NK cells ↑	NS
			intraperitoneal		NS	Treg cells ↑	Treg cells↑	B cells ↓, G-MDSC & NK cells ↑	B cells & Macrophages ↓, G-MDSC ↑↑ & M-MDSC ↑
		whole uterus (endometrium and myometrium)	subcutaneous		NS	NS	NS	B cells ↓, G-MDSC & NK cells ↑	NS
			intraperitoneal		NS	NS	NS	B cells ↓, G-MDSC & NK cells ↑	B cells & Macrophages ↓, G-MDSC ↑ & M-MDSC ↑

G-MDSC, granulocytic myeloid-derived suppressor cells; NK, natural killer; M-MDSC, monocytic myeloid-derived suppressor cells. ↓:significantly decreased; ↑: significantly increased; NS, no significance.

## Discussion

Our study showed different endometriosis modeling methods result in different pathological and immunological features. Ovariectomy with estrogen supplement, solely endometrium fragments, and intraperitoneal injection are more suitable for the pathological and immunological studies in endometriosis, which are important for mechanistic studies and immunotherapy development.

Endometriosis can appear as black, dark-brown, dark blue, red, white, yellow, or non-pigmented lesions on the ovaries, peritoneum, uterus, and even extragenitalis (such as bowels and bladder) and extrapelvic sites (such as the abdominal wall, kidneys, and lung) (30). The typical histopathology of endometriosis usually contains endometriotic glands and stroma and sometimes also presents with hemosiderin deposits and fibrosis. In endometriosis mouse models, the experimental endometriotic lesions show some similar histological characteristics of the human ectopic lesions, including endometrial glands, stroma, and/or cysts. There are various mouse models in the literature of endometriosis research, and the generation of these models involves some important factors regarding the estrus cycle staging and transplantation methods. There are several methods for estrus cycle synchronization, which may have their own advantages and limitations. For example, transfering urine-soaked male bedding to the female cage while other recipient rodents are ovariectomized and treated with estrogen to maintain lesion growth (31, 32). If we use intact cycling animals, we should monitor the estrus cycle daily by examination of vaginal cytology (33). It is more physiologically relevant but complicated to do on a large scale and sometimes fails to achieve cycle synchronicity (34). Since sex hormones will influence the immune system, mice in various estrus cycle stages will have different immune responses. Alternatively, giving exogenous E_2_ supplements to ovariectomized animals can obtain uniform hormonal levels among many animals, and it is easier to synchronize the hormone levels in large amounts. In the mouse model of endometriosis by OVX with E2 supplement, the epithelium, stroma, vasculature, estrogen receptor β, and macrophages are all present in the endometriotic lesions that exhibit similarities to those from endometriosis patients (35). In our mouse model of endometriosis by male urine-soaked transfer bedding, the presence of endometrial glands, stroma, and macrophages was also demonstrated; thus, in terms of growth, it is comparable in two synchronization methods (34). However, exogenous E_2_ supplement influences the development of endometriosis itself (6). E2 is a major factor related to weight gain and endometrial hyperplasia in women (36), so it may also contribute to the significant body weight increase in mice. In the present study, the lesion wall area, average lesion thickness, and average endometrium thickness were higher in the TB group than in the OVX group. However, we found a higher proliferative activity in the epithelium cells of the OVX group, which may contribute to the difference in lesion thickness. It also has a higher transplantation success rate in the OVX group. To sum up, OVX and exogenous E_2_ supplement are likely to achieve the endometriosis model at ease and in success.

The development of endometriosis is a complex process with chronic inflammation as the central pathophysiology of the disease; previous studies have demonstrated an elevated number of dysfunctional immune cells along with a higher concentration of the pro-inflammatory cytokines in the peritoneal cavity (37). However, there was a study that showed that endometriosis immunology exhibits features of cells that are capable of evading immunosurveillance (38), so the pathogenesis of endometriosis is associated with the capacity of endometrial cells to counteract the ongoing local immunological response. Successful immune escape of the retrograded endometrial cells from immune surveillance is one of the key steps in the early development of endometriosis, probably every time during each menstrual cycle. Since women are rarely diagnosed in the early stage and because of some ethical considerations, little is known about the pathogenic progress and immune changes in the establishment of endometriosis in an early stage. At the time of infections, immune cells including macrophages will provide the first line of defense in the innate immune response and release cytokines to recruit other immune cells, like neutrophils and monocytes (39). A decrease in NK cell cytotoxicity (40) and enhanced activation of peritoneal macrophages (41) also appear in women with endometriosis. Treg cells play an important role in suppressing and controlling various immune responses and are highly disturbed in ectopic peritoneal lesions in women suffering from endometriosis (42). MDSCs are a heterogeneous population of myeloid cells that expand under pathological conditions and have immunosuppressive properties by suppressing T-cell responses (43). Our group previously found that MDSCs were significantly increased in the peripheral blood of women with endometriosis, and a similar immune response was found in the endometriosis mouse model also (20). Accumulation of peritoneal fluid and/or blood MDSCs and Tregs in women with endometriosis and experimental mouse models has been reported in other studies (44–46). In previous animal studies, both separated endometrium from the uterus and whole uterus tissue were used, and the quantity of challenged endometrium positively correlated with the extent of induced endometriosis without severe side effects (19). In the present study, both the EmIP and WIP groups caused similar changes in immune cells when compared to the control, but only the EmIP group caused a significant increase of Treg cells compared to the WIP group and control group in the spleen and lymph node. In other studies, an elevated or unchanged number of macrophages was found in the endometriosis mouse model (47, 48); these discrepant results may be due to the timing of sample collection, small sample size, and different methods of detection, and also the studies were not compared in the same settings and controls. In our study, we found that the purity of endometrium plays an important role in the early immune response of mouse endometriosis model.

Endometriosis is a chronic inflammatory condition because the endometrium is repeatedly shed and retrograded through the oviduct to the peritoneal cavity in every menstrual cycle. Therefore, the immunological responses of the endometrium are periodic as in the menstrual cycle. In each menstrual cycle, the retrograded endometrium occurs mostly during the period of menses. When menses cease, the retrograded endometrium is expected to cease as well. Therefore, the immune response to the retrograded endometrium in the peritoneal cavity is actually an acute immune response, while the immune response to the migrated and invaded ectopic endometrium in the peritoneal lining is a chronic immune response that is rather too late and refractory to be targeted by the pharmaceutical therapy. Therefore, it is vitally important to study the early immune profiles. Based on our previous study, the early immune response of endometriosis mainly appeared within 24 h (20), so in our study, we focused on early immune profiles in a such short period.

However, we compared the endometriosis mouse model by intraperitoneal injection (20, 49) with the model by subcutaneous transplantation (50) with some minor modifications. Both models show histological characteristics of human endometriosis, including the presence of glands and stroma. The subcutaneous transplantation model is commonly used to study the effects of therapeutic drugs and chemicals. The intraperitoneal injection model is commonly used to study immunoreaction in a peritoneal microenvironment, but there are limitations. The established endometriotic lesions tend to be very small and embedded in the abdominal organs, making them difficult to retrieve; moreover, it is not easy to monitor lesion growth without opening the abdomen. In the present study, obvious immunoreaction can be detected in the peritoneal fluid in the intraperitoneal injection model but not in the subcutaneous transplantation model, suggesting the endometriotic lesions outside the peritoneal cavity may not be sufficient to induce as strong an immune response as inside the peritoneal cavity. In addition, in the intraperitoneal injection model, MDSCs significantly increased in both peripheral blood peritoneal microenvironments, which is similar to endometriosis in humans. Therefore, we speculate that the MDSCs in the endometriosis mouse model were motivated from the bone marrow, expanded in peripheral blood, and recruited into the ectopic endometrial site intend to suppress immune cell functions and promote endometriotic development and growth in the peritoneal cavity. Some of the endometrial tissue fragments do escape apoptosis, invade contacted surface, and acquire a vascular supply. Taken together, intraperitoneal injection is a better model to study the immunological studies of endometriosis in mice.

## Conclusions

Different endometriosis modeling methods result in different pathological and immunological features. Ovariectomy with estrogen supplement, solely endometrium fragments, and intraperitoneal injection are more suitable for the pathological and immunological studies of endometriosis in mice, which are important for mechanistic studies and immunotherapy development.

## Data availability statement

The raw data supporting the conclusions of this article will be made available by the authors, without undue reservation.

## Ethics statement

The animal study was reviewed and approved by the government of the Hong Kong Special Administrative Region Department of Health.

## Author contributions

YH, HX, and CW designed the study. YH, BL, SH, and RZ performed the experiments. YH wrote the manuscript. HX, JC, and CW revised the article. All authors contributed to the article and approved the submitted version.

## Funding

This research was supported by the Health and Medical Research Fund (08190886) from the Food and Health Bureau, The Hong Kong Obstetrical & Gynaecological Trust Fund 2019 from the Hong Kong Society of Obstetricians and Gynaecologists, and Academic Equipment Grant (3029876) and Direct Grant (2017.044) from The Chinese University of Hong Kong.

## Conflict of interest

The authors declare that the research was conducted in the absence of any commercial or financial relationships that could be construed as a potential conflict of interest.

## Publisher’s note

All claims expressed in this article are solely those of the authors and do not necessarily represent those of their affiliated organizations, or those of the publisher, the editors and the reviewers. Any product that may be evaluated in this article, or claim that may be made by its manufacturer, is not guaranteed or endorsed by the publisher.
